# Novel hollow biodegradable microneedle for amphotericin B delivery

**DOI:** 10.1002/mco2.321

**Published:** 2023-07-06

**Authors:** Sina Azizi Machekposhti, Sachin Kadian, Lyndsi Vanderwal, Shane Stafslien, Roger J. Narayan

**Affiliations:** ^1^ Joint UNC/NCSU Department of Biomedical Engineering North Carolina State University Raleigh North Carolina USA; ^2^ Department of Coatings and Polymeric Materials North Dakota State University Fargo North Dakota USA

Dear Editor,

Several approaches have been previously described for incorporating drugs within polymer microneedles.^1^, ^2^, ^3^, ^4^, ^5^ Our previous study^1^ aimed to deliver amphotericin B by biodegradable solid microneedles. In this approach, amphotericin B was mixed with Gantrez® AN 119 BF; the mixture of Gantrez® AN 119 BF and amphotericin B was left at room temperature for approximately 2 weeks to be solidified in the shape of microneedle. Although the approach was successfully used with amphotericin B, it may not be suitable for some drugs.^2^ For example, the mechanical properties of some polymers may be lowered after being combined with certain drugs. Other studies involve coating microneedles with drugs; however, there may be a limit to the dosage that can be applied using the coating approach.^4^, ^5^

To determine the volume of the amphotericin B loaded in each hollow microneedle, the amphotericin B‐loaded microneedles were broken in tubes and dissolved in dimethyl sulfoxide:methanol; the amphotericin B concentration was determined by high‐performance liquid chromatography. High‐performance liquid chromatography indicated that there were 2.00 ± 0.08 mg of amphotericin B in each hollow microneedle.

Laser confocal microscopy was used to assess the height, base diameter, and hollow features of the hollow microneedle. Figure 1A shows the length associated with the outer layer of a hollow microneedle, Figure 1B shows the length associated with the hollow part of a hollow microneedle, and Figure 1C shows the 3D image of a hollow microneedle. The height of the microneedle outer layer and microneedle base diameter are 858.03 and 424.82 μm, respectively. For the hollow part of the microneedle exhibits height, base diameter, and volume values of 653.75, 366.61 μm, and 2.3 × 10^7^ μm^3^, respectively. Figure 1B shows the hollow part of a needle that can be loaded with amphotericin B powder or other drug powders. Since the base diameter measurement difference between Figures 1A and B is 58.21 μm, the thickness of the Gantrez^®^ AN 119 BF shell is 29.1 μm. Figure S2C shows that the tip of the loaded needles is not yellow; as such, the microneedle tip is not loaded with amphotericin B. Figure 1C is the confocal laser microscopy image of the microneedle, which shows the sharp tip and the uniform level of roughness throughout the microneedle surface.

**FIGURE 1:**
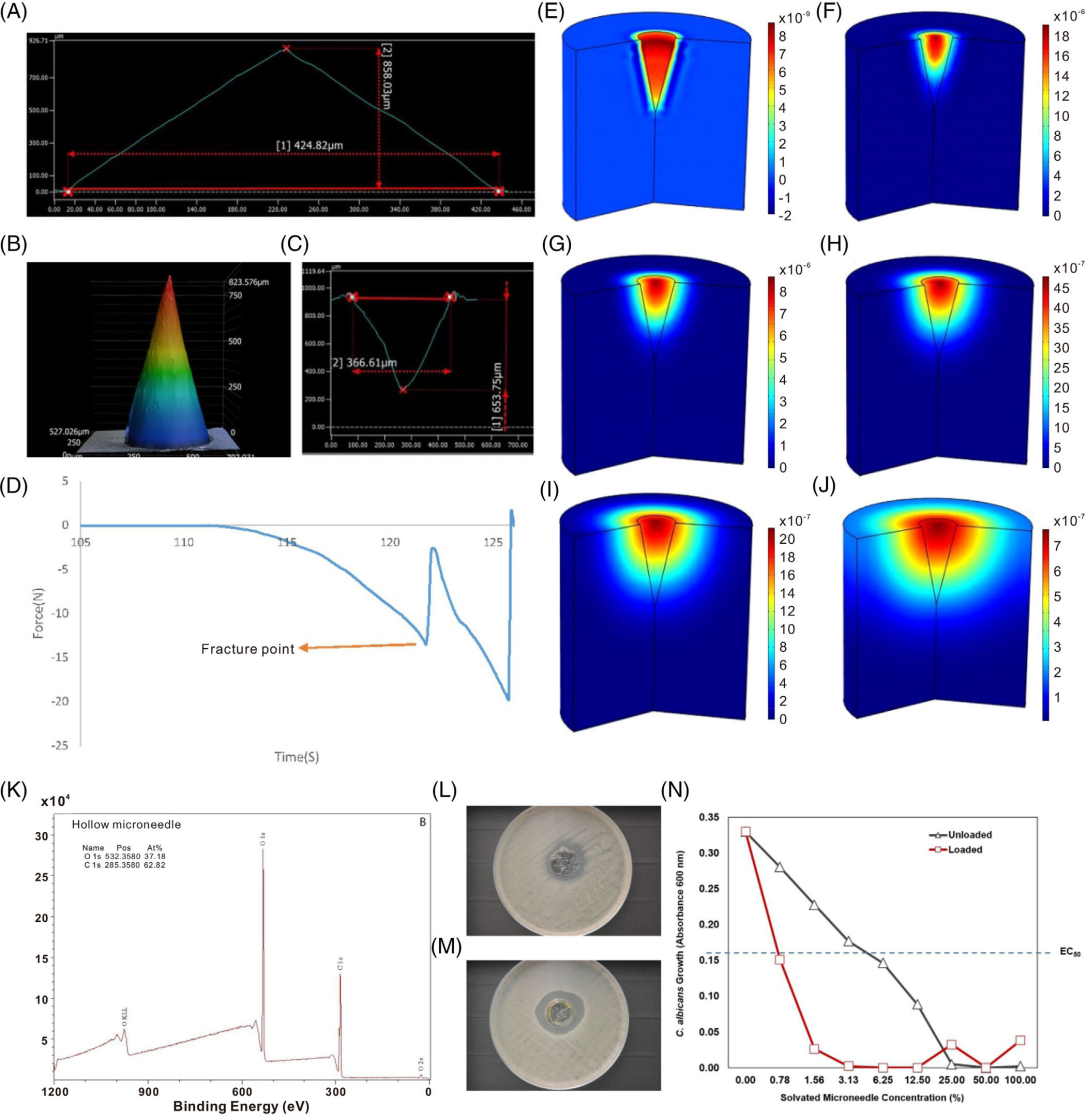
(A) Laser confocal microscope measurement showing the length associated with the outer layer of an unloaded hollow microneedle. (B) Laser confocal microscope measurement showing the length associated with the hollow part of the hollow microneedle. (C) Laser confocal microscope image of a hollow Gantrez® AN 119 BF microneedle showing the external morphology of the microneedle. (D) Electroforce 3100 (Bose Corporation, Framingham, MA) force–time data showing the fracture point of needle under compressive loading. The fracture force per microneedle was 0.6 N. COMSOL Multiphysics® 6.0 data for drug diffusion of amphotericin B in skin after microneedle insertion into skin (E: 0 h, F: 2 h, G:6 h, H: 12 h, I:24 h, and J: 48 h). The lateral surface area of the cylinder boundary condition is considered “symmetric,” the top circle is considered “no flow,” and the below circle surface area of the cylinder is considered “open boundary.” The effective diffusion coefficient of amphotericin B in the skin is 7.71 × 10^−13^. Concentration dimension is (mol/m^3^). (K) Survey scan X‐ray photoelectron spectrum from hollow microneedle. The survey scan indicated that the Gantrez® AN 119 BF hollow microneedle contains 37.18% O and 62.82% C. Images of *C. albicans* agar diffusion results for the unloaded hollow microneedle with a 28 mm ZOI (L) and the amphotericin B‐loaded hollow microneedle with a 33 mm ZOI (M). (N). Solution growth profile of *C. albicans* (600 nm) as a function of solvated microneedle concentration for both amphotericin B‐loaded microneedles (red line) and unloaded hollow microneedles (black line).

Previous studies have used compression studies to determine the failure force of the microneedles.^5^ Boehm et al.^6^ previously showed that the Young's modulus and hardness of another Gantrez^®^ AN material, Gantrez^®^ AN 169 BF, were 6.34 ± 0.41 GPa and 216.39 ± 2.87 MPa (mean ± standard deviation). Nanoindentation of Gantrez^®^ AN 119 BF provided a Young's modulus value of 7.01 ± 0.33 GPa and a hardness value of 251 ± 9.56 MPa; this Young modulus value should be sufficient for human skin penetration.^7^ Figure 1D shows the fracture curve of 25 needle array obtained from a Bose Electroforce instrument for needles under compression; failure at −15 N was noted (the minus sign indicates compressive loading); the fracture force per microneedle was 0.60 N. This test was repeated with an additional three hollow microneedle devices; the fracture force per hollow microneedle was noted as 0.60 ± 0.03 N.

To understand how the release of the drug in the skin, a drug diffusion simulation study was performed using COMSOL Multiphysics^®^ 6.0. The amphotericin B diffusion coefficient in skin interstitial fluid (plasma) is 2.57 × 10^−8^, and the effective diffusion coefficient is 7.71 × 10^−13^. Drug release starts after the Gantrez^®^ AN 119 BF microneedle wall is dissolved in the skin, which takes approximately 20 min. This information was used to simulate drug diffusion in the skin. Figures 1E–I and J show the drug diffusion process after 0 h, 2 h, 12 h, 24 h, 2 days, and 4 days after microneedle insertion, respectively. This simulation may be helpful to understand drug diffusion in the skin after microneedle insertion.

Figure 1K shows the XPS spectrum from the hollow microneedle. The survey scan indicated that the Gantrez^®^ AN 119 BF hollow microneedle contains 37.18% O 1s and 62.82% C 1s. The amphotericin B‐loaded hollow microneedle showed the same XPS result as Gantrez^®^ AN 119 BF hollow microneedle because no amphotericin B appears on the surface of the hollow Gantrez^®^ AN 119 BF microneedle. The survey spectrum shows the presence of only C, O, and N, which correspond to the polymer composition. The spectra do not show other elements (e.g., no toxic or metallic impurities).

The skin penetration of the as‐prepared biodegradable hollow microneedle array was assessed ex vivo using porcine skin. For this study, the fresh porcine skin was purchased from the supermarket and cleaned with an alcohol swab. Then, the cleaned skin was firmly fixed on a board and manually punctured (Figure S3A) with our as‐prepared biodegradable hollow microneedle. Next, in order to show the skin penetration characteristics of the microneedle array, 0.4% Trypan Blue solution was applied to microneedle‐treated skin for better visualization of the pores. It can be observed from Figures S3B and C that the developed biodegradable hollow microneedle array can readily penetrate the porcine skin for drug delivery application.

Figure 1L shows that the *C. albicans* zone of growth inhibition (ZOI) for the unloaded hollow microneedle device was 28 mm; in contrast, the ZOI for the amphotericin B‐loaded hollow microneedle device was 33 mm (Figure 1N). The larger ZOI observed for the amphotericin B‐loaded hollow microneedle indicates that *C. albicans* is more susceptible to amphotericin B‐loaded microneedle device than its unloaded microneedle device due to the diffusion of amphotericin B into the nutrient agar; the unloaded microneedle has previously been shown to possess some antifungal activity.^4^

Figure 1N shows the solution growth profiles of *C. albicans* as a function of solvated hollow microneedle concentration for both amphotericin B‐loaded and unloaded Gantrez^®^ AN 119 BF hollow microneedle devices. Both microneedle variants were fully solvated in PBS (1×) to characterize their ability to prevent planktonic *C. albicans* growth. A 1:2 dilution series of the fully solvated microneedle devices in nutrient growth media was created and evaluated in this study. The 3.13% solvated amphotericin B‐loaded microneedle concentration completely inhibited the growth of *C. albicans*. In contrast, for the unloaded microneedle devices, less than 50% *C. albicans* growth inhibition was observed for the 3.13% concentration; complete growth inhibition was only observed at the three highest concentrations assessed (25, 50, and 100%). When taken in combination with the agar diffusion results, the comparative solution growth profiles demonstrate a higher antifungal activity for the amphotericin B‐loaded microneedles than for unloaded microneedles. Table S1 provides the MIC/E_50_ of both amphotericin B‐loaded and unloaded hollow microneedle devices where MIC denotes the percent concentration that completely inhibited growth, and EC_50_ denotes the solvated microneedle concentration that reduced growth by 50% (i.e., half maximal effective concentration); the MIC/EC50 values were 25.00/6.25 for the amphotericin B‐loaded microneedle devices and 3.13/0/78 for the unloaded microneedle devices.

This study proposes a novel type of biodegradable hollow microneedle; the limitation of this technique is that the drug must be available in a uniform powder. The unloaded hollow microneedle was fabricated at room temperature; it was then loaded with the drug in less than one hour by simultaneous vacuuming and shaking. This technique can be extended for use with other kinds of biodegradable polymers and drugs in powder form.

## AUTHOR CONTRIBUTIONS

*Methodology, investigation, writing‐original draft preparation, and writing—review and editing*: S. A. M, L. V., and S. S. Writing—review and editing, supervision, project administration, and funding acquisition: R. J. N. All authors have read and approved the final manuscript.

## CONFLICT OF INTEREST STATEMENT

The authors declare no conflict of interest.

## ETHICS STATEMENT

Institutional Review Board Statement and Informed Consent Statement are not applicable.

## Supporting information

Supporting information

## Data Availability

The datasets generated during and/or analyzed during the current study are available from the corresponding author on reasonable request.
