# Supplementary material for: Novel hollow biodegradable microneedle for amphotericin B delivery
Source: MedComm (2020). 2023 Jul 6;4(4):e321. doi: 10.1002/mco2.321 (PMC10323634; doi:10.1002/mco2.321)
Supplement: Supplementary file 1 — Supporting information [file MCO2-4-e321-s001.docx]

**Supplementary Material**

**Novel hollow biodegradable microneedle for amphotericin B delivery**

Sina Azizi Machekposhti^1^, Sachin Kadian^1^, Lyndsi Vanderwal^2^, Shane Stafslien^2^, and Roger J. Narayan^1^*

^1^Joint UNC/NCSU Department of Biomedical Engineering, North Carolina State University, Raleigh, NC, USA; sazizim@ncsu.edu; [skadian@ncsu.edu](mailto:skadian@ncsu.edu); roger_narayan@unc.edu

^2^Coatings and Polymeric Materials, North Dakota State University, Fargo, ND, USA; lyndsi.vanderwal@ndsu.edu; [shane.stafslien@ndsu.edu](mailto:shane.stafslien@ndsu.edu);

Corresponding Author: roger_narayan@unc.edu

**Materials and Methods**

**Hollow microneedle fabrication and drug loading**

The fabrication process is performed based on micromolding with a master structure. The master structure was processed using a Model S130 digital light processing (DLP)-based additive manufacturing instrument from Boston Micro Fabrication (Maynard, MA). This instrument provides a layer height of 5 $\mu$m, which correlates with the vertical precision, and a horizontal precision of 2 $\mu$m. The master structure was processed from a biocompatible resin named BIO, which is distributed by Boston Micro Fabrication (Maynard, MA). This yellow transparent resin is a mixture of two methacrylate oligomers, diphenyl(2,4,6-trimethylbenzoyl)phosphine oxide, which acts as a photoinitiator, and lauryl methacrylate, which acts as a reactive diluent. Per the manufacturer, the water absorption and contact angle values associated with this material are 0.69%, and 50-70^o^, respectively. Per the manufacturer, the material has passed several ISO studies, including the ISO 10993-10: 2010, ISO 10993-12: 2012, and ISO 10993-2: 2006 skin irritation tests; ISO 10993-10: 2010, ISO 10993-12: 2012, and ISO 10993-12: 2006 skin sensitization tests; ISO 10993-4: 2017 in vitro hemolytic test; ISO 10993-11: 2017, ISO 10993-12: 2012, and ISO 10993-2: 2006 pyrogen tests; ISO 10993-5: 2009 and ISO 10993-12: 2012 in vitro cytotoxicity tests; and ISO 10993-2: 2006 acute systemic toxicity test ^1^. After additive manufacturing-based processing, the master structure was post cured with a Form Cure lamp (λ=405 nm) (Formlabs Inc., Somerville, MA) for 10 minutes at 45 ^o^C. The microneedle height, base diameter, and microneedle-to-microneedle spacing were 900 $\mu$m, 430 $\mu$m, and 700 $\mu$m, respectively. The molds were fabricated by a micromolding technique from these master structures.

A polydimethylsiloxane master structure was fabricated to create the hollow components of the microneedle. The polydimethylsiloxane master structure was replicated from a master structure that was printed on the S130 digital light processing (DLP)-based additive manufacturing instrument. The hollow microneedle height, base diameter, and spacing were 700 $\mu$m, 230$\mu$m, and 700 $\mu$m, respectively. The master structure was used to fabricate a mold from PMC-746 polyurethane rubber compound (Reynolds Advanced Materials, Lower Macungie Township, PA). The polydimethylsiloxane master structure was replicated in a furnace at 65^o^ C for two days. The polydimethylsiloxane microneedle was then carefully extracted. The polydimethylsiloxane master structure was placed in back of the polydimethylsiloxane mold to make the hollow part of the hollow microneedle; the schematic is shown in Figure S1. The stickness of the polydimethylsiloxane and Gantrez® AN 119 BF (Ashland Global Specialty Chemicals Inc., Wilmington, Delaware) was negligible. Liquid Gantrez^®^ AN 119 BF was poured into the mold, followed by simultaneous vacuuming and sonicating; the resultant material was left in the ambient environment for water evaporation. After approximately two weeks, all of the extra water evaporated; the Gantrez^®^ AN 119 BF microneedle was extracted from the mold. For loading the drug in the bores, amphotericin B powder was added to the base of the hollow microneedles and then shaken and vacuumed simultaneously. The vacuuming pressure was 25 psi, and the shacking speed was 120 rpm; vacuuming and shaking were applied for 40 minutes. Finally, excess amphotericin B powder was gathered, the back part of the hollow microneedle was cleaned, and the back part of the hollow microneedle was closed by an acrylate-based adhesive. The hollow microneedle microstructure was assessed using a SM-3TZZ-54S-10 ​M Amscope microscope (United Scope LLC, Irvine, CA).

A Dimatix material printer was also considered for loading the hollow microneedle with amphotericin B. The Dimatix DMP-2831 material printer (Fujifilm Dimatix, Santa Clara, CA, USA) and the DMCLCP-11610 inkjet cartridge (Fujifilm Dimatix, Santa Clara, CA, USA) were used for processing amphotericin B. The cartridges were loaded with 1 ml of amphotericin B solution using a syringe and a blunt-tipped needle. The DMCLCP-11610 inkjet cartridge ejects droplets with 10 pL volume ^2^. To make the cartridge solution, amphotericin B dissolved in various solvents such as ethanol, methanol, isopropyl alcohol, and acetic acid, separately. The hollow microneedle was placed on the Dimatix material printer stage vertically, with the tip side placed in the downward orientation. The location of each microneedle center was inputted manually into the Dimatix material printer software. The center of the left top microneedle was found with the printer camera and set as the starting printing point. The cartridge was placed in the appropriate location in the Dimatix material printer and used for printing. Both printer head and stage were sterilized with isopropyl alcohol. The above-mentioned approach was repeated using various solutions of amphotericin B with different hollow microneedles.

**SEM Imaging**

An S-3200 scanning electron microscope (Hitachi, Tokyo, Japan) was used to assess the microscale features of the microneedle. The hollow microneedles were broken with a tweezer to facilitate the assessment of the microneedle wall thickness.

**Mechanical Properties**

To assess the Young’s modulus and hardness values of the Gantrez^®^ AN 119 BF material, nanoindentation was performed using a Hysitron TI980 Triboindenter (Bruker Corporation, Billerica, MA) with a 3-sided pyramidal (Berkovich) tip at six locations. The samples are glued and placed on the device stage. The maximum force used in this study was 1000 μN; the loading time, dwell time at maximum load, and unloading time were 20 s, 10 s, and 20 s, respectively. The load was applied in the normal orientation to the Gantrez^®^ AN 119 BF material. The unloading curves were analyzed using the Oliver-Pharr ^3^ approach to determine the hardness and Young’s modulus values.

An Electroforce 3100 instrument (TA Instruments, New Castle, DE) was used to evaluate the fracture properties of the hollow microneedle under compressive loading. The hollow microneedle was placed on the down platen and held by double-sided tape in a perpendicular orientation. The top platen was actuated downward in three steps. The first step started with a load of 0.005N/s to a load of -0.015 N. This step was used to bring the hollow microneedle in contact with the probe. The probe dwelled in place for 10 seconds. The last waveform was a displacement at a rate of 0.0025 mm/s to reach a 1.5 mm displacement. The hollow microneedles fractured prior to the 1.5 mm displacement.

**Laser Scanning Confocal Microscopy**

A VK-X250 3D laser scanning microscope (Keyence, Tokyo, Japan) was used to determine the height, base diameter, tip diameter, and hollow volume measurements of the hollow microneedle. This microscope contains laser confocal optics, which enable field depth measurements to be made. The microscope contains a white light source and a 408 nm wavelength laser light for obtaining height information and images. In this instrument, the laser was rastered in an XY pattern; the Z-direction steps were 0.5 nm.

**Loaded drug determination by high-performance liquid chromatography**

To understand the amount of the drug in each microneedle device, the drug-loaded microneedles were broken in a tube; a sufficient amount of DMSO:methanol (1:1) was added to dissolve the microneedles and prepare a liquid solution. High performance liquid chromatography studies to determine the drug concentration was performed by an external provider (Fungus Testing Laboratory, UT Health San Antonio, San Antonio, TX). Since the solution volume is known, drug weight is calculated in every hollow microneedle; this measurement was repeated five times. A Shimadzu high performance liquid chromatography instrument (Kyoto, Japan) was used to evaluate the amount of drug in each hollow microneedle; a Luna 5µm 150 × 4.6 mm C18 column (Phenomenex, Torrance, CA) was used in this study. The mobile phase that was used for the chromatographic separation of amphotericin B was N,N,N′,N′-tetramethyl ethylenediamine:acetonitrile (65:35 v/v). A 1.1 ml/min isocratic flow rate was used; the injection volume was 50 μl. The retention time for amphotericin B was 4.497 min; the detection measurement was performed using an ultraviolet light detector with a wavelength of 406 nm.

**XPS**

X-ray photoelectron spectroscopy was used to determine the chemical composition and the presence of impurities on the microneedle surface ^4,5^. A SPECS instrument with a PHOIBOS 150 Hemispherical Analyzer (SPECS Surface Nano Analysis GmbH, Berlin, Germany) was used for the X-ray photoelectron spectroscopy measurements. Measurements were obtained from both hollow microneedle tips and base sides. Data was acquired from the amphotericin B powder, unloaded hollow microneedle, and amphotericin B-loaded hollow microneedle.

**Drug Diffusion Simulation**

To simulate the drug diffusion functionality in the skin, the bulk diffusion coefficient was determined experimentally based on an approach that was described in an earlier study ^6^. This method was validated by determining the diffusion coefficient of the known materials like sucrose in water. By determining the diffusion coefficient of amphotericin B in blood plasma, the effective diffusion coefficient was calculated by knowing the skin porosity. Equation 1 shows the correlation between the diffusion coefficient and effective diffusion coefficient ^7^.

| $D_{eff}=\varepsilon D$ | (1) |
| --- | --- |

where $\varepsilon$ is skin porosity; the average value was determined by earlier study($\varepsilon=2E-5$) ^8^. $D_{eff}$ is effective diffusion coefficient defined in porous media, and D is diffusion coefficient between two liquids out of porous media. Drug release starts after the hollow wall of the microneedle is dissolved in the skin. The wall thickness is approximately 0.04 mm; the wall takes approximately 0.4 min to be dissolved completely in pure plasma. This wall takes approximately 20 minutes to be dissolved in skin interstitial fluid.

**Antimicrobial study:**

An agar diffusion and solution activity assessment were performed with the pathogenic yeast *Candida albicans* (ATCC 10231, American Type Culture Collection, Manassas, VA); the antimicrobial properties of the amphotericin B loaded hollow microneedle were evaluated as described previously ^9^. Amphotericin B-loaded and unloaded hollow microneedles were dissolved/solvated prior to agar diffusion testing. The fully solvated amphotericin B-loaded and unloaded Gantrez^®^ AN 119 BF hollow microneedles were dispensed in 30 mm void area that was cut into the center of Sabouraud dextrose agar to prevent materials from spreading across the agar surface during thermal/hydration-induced gelation. For solution activity assessments, the hollow microneedle samples were solvated in 3 mL of 1 x phosphate-buffered saline; the solvated fraction was considered 100% strength/concentration. A 1:2 dilution series of the 100% strength solution was prepared in 1X yeast nitrogen broth + dextrose to achieve a solution strength/concentration range of 100% down to 0.78%. The dilution series was subsequently inoculated with *Candida albicans*; end point growth at 24 hours was quantified via solution absorbance measurements (λ=600 nm).

**Results from optical microscopy imaging:**

The hollow microneedle was fabricated using a micromolding technique. One challenge associated with fabrication of the hollow microneedle is obtaining microneedles with a sufficiently thick wall; if the wall thickness is insufficient, then loading the microneedle with a drug may lead to fracture. Figure S2 (A) shows the first type of microneedles; several cracks were noted in the hollow microneedles and base. The base cracks are attributed to exposure to the ultrahigh vacuum in the scanning electron microscope.

In subsequent efforts, attempts were made to create wider and more symmetrical hollow microneedle walls by optimizing the viscosity of liquid Gantrez^®^ AN 119 BF. Some microneedles are broken for imaging; the scanning electron microscopy images showed a satisfactory thickness for each side (Figure S2 (B)). Cracks are present in both Figure S2 (A) and Figure S2 (B). Pure solidified Gantrez^®^ AN 119 BF was noted to crack in vacuum pressure higher than 1.72 bar; the SEM images contain cracks that are not evident prior to placement in a vacuum environment.

Figure S2 (C) and (D) show an image of an amphotericin-B-loaded hollow microneedle that was obtained using an optical microscope. Figure S2 (C) shows the hollow microneedles on a blue substrate to enhance visualization of the microneedles, and Figure S2 (D) shows the hollow microneedles on a colorless substrate to show how the amphotericin B powder fills the microneedle bores.

**References**

1. https://bmf3d.com/micro-3d-printing-materials/.

2. Azizi Machekposhti S, Zhang B, Sachan R, Vanderwal L, Stafslien SJ, Narayan RJ. Patterned surfaces with the controllable drug doses using inkjet printing. Journal of Materials Research. 2021;36(19):3865-3876.

3. Yan W, Pun CL, Simon GP. Conditions of applying Oliver–Pharr method to the nanoindentation of particles in composites. Composites Science and Technology. 2012;72(10):1147-1152.

4. Pop-Georgievski O, Kubies D, Zemek J, et al. Self-assembled anchor layers/polysaccharide coatings on titanium surfaces: a study of functionalization and stability. Beilstein journal of nanotechnology. 2015;6(1):617-631.

5. Pandey P, Mitra MD, Shukla S, Narayan RJ. Organotrialkoxysilane-functionalized mesoporous Pd–Ni nanocatalyst for selective hydrazine decomposition and sensing. MRS Communications. 2021;11:78-85.

6. Linder PW, Nassimbeni LR, Polson A, Rodgers AL. The diffusion coefficient of sucrose in water. A physical chemistry experiment. Journal of Chemical education. 1976;53(5):330.

7. Khaled A-R, Vafai K. The role of porous media in modeling flow and heat transfer in biological tissues. International Journal of Heat and Mass Transfer. 2003;46(26):4989-5003.

8. Mitragotri S. Modeling skin permeability to hydrophilic and hydrophobic solutes based on four permeation pathways. Journal of Controlled Release. 2003;86(1):69-92.

9. Azizi Machekposhti S, Nguyen AK, Vanderwal L, Stafslien S, Narayan RJ. Micromolding of Amphotericin-B-Loaded Methoxyethylene–Maleic Anhydride Copolymer Microneedles. Pharmaceutics. 2022;14(8):1551.

| Supplementary Table 1: The MIC and EC_50_ values for solvated amphotericin B loaded and unloaded hollow microneedles, which were tested against the pathogenic yeast *C. albicans*. |
| --- |
| \| **Hollow Microneedles/Compound** \| **MIC/EC_50_ (% Concentration)** \| \| --- \| --- \| \| unloaded \| 25.00/6.25 \| \| amphotericin B-loaded \| 3.13/0.78 \| |

| 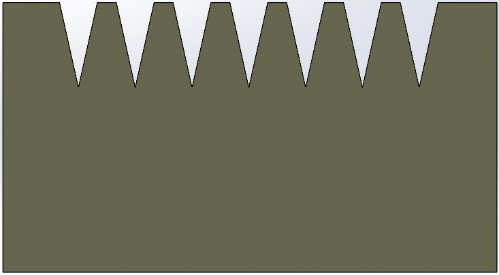 | 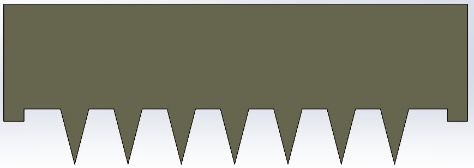 | 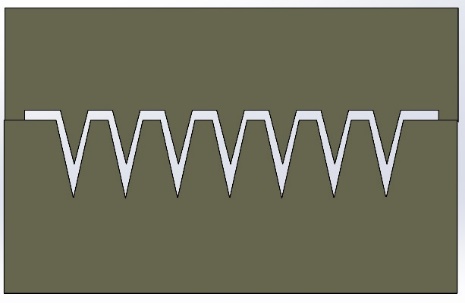 |
| --- | --- | --- |
| Fig. S1. The left image shows the PDMS mold, and the middle image shows the PDMS microneedles. PDMS microneedles are placed on top of the mold; each microneedle went through the mold bore (the right image). The gap between mold and microneedles was filled with the liquid Gantrez® AN 119 BF material. To push the liquid Gantrez® AN 119 BF material to the gap, a vacuum pump was used. Then the microneedles were left at room temperature for 10 days for evaporation. Finally, hollow Gantrez® AN 119 BF microneedles were removed carefully from the mold, and the PDMS microneedles were removed carefully from the hollow Gantrez® AN 119 BF microneedles. | | |


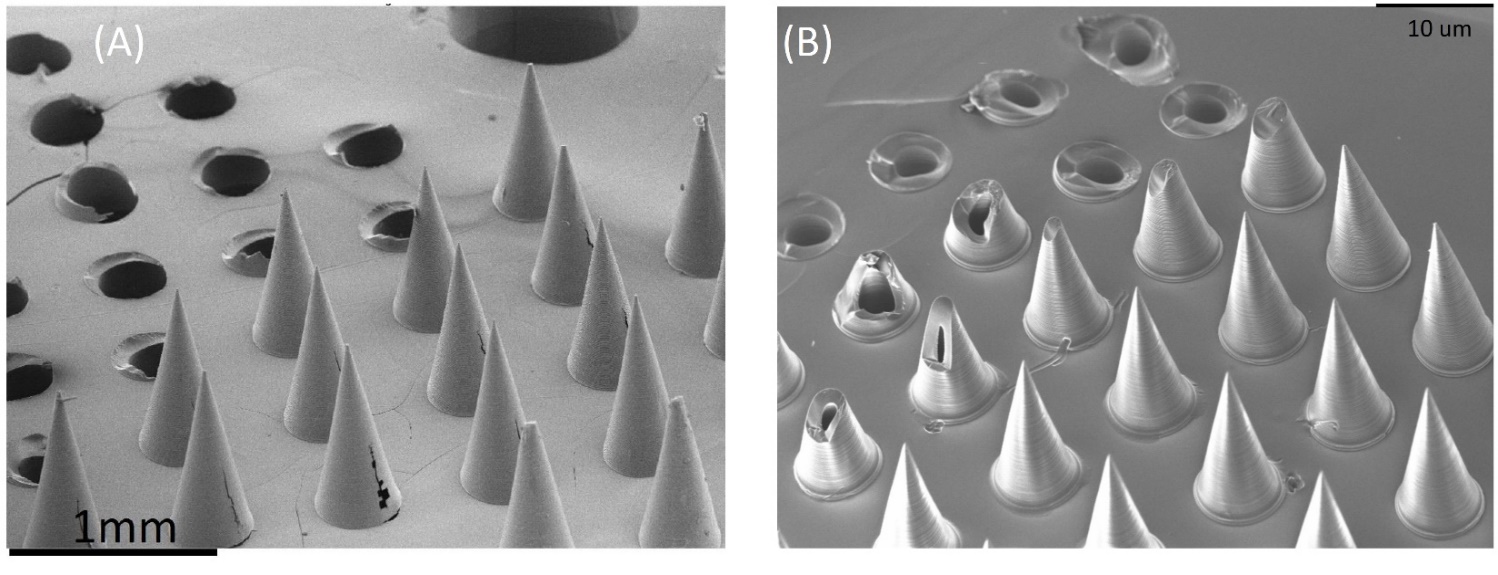


| 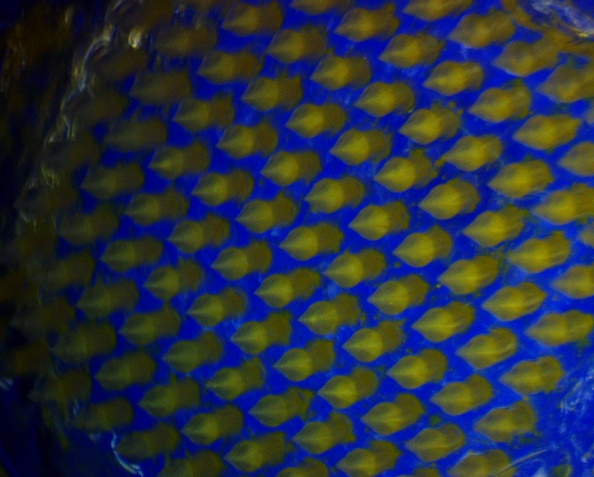  C | 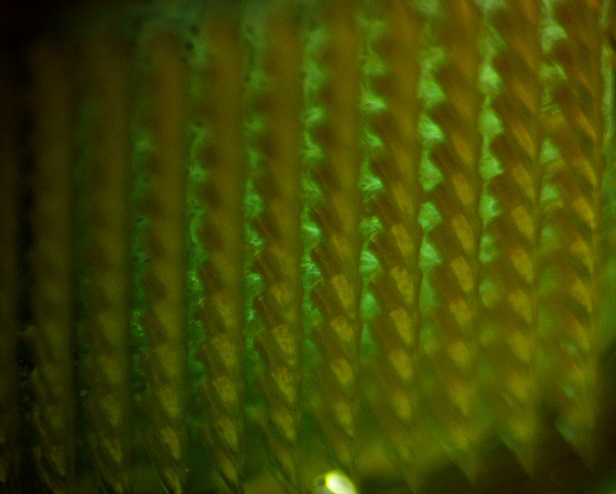  D |
| --- | --- |

Fig. S2. (a) Scanning electron micrograph of the unloaded pure Gantrez^®^ AN 119 BF microneedle. An asymmetrical microneedle wall thickness was noted. Cracks at the base of the microneedles were attributed to the exposure to vacuum in the scanning electron microscope. (b) The symmetrical wall thickness is appropriate for loading the hollow microneedle with amphotericin B, and also results in higher mechanical strength of the microneedles for piercing the skin. Optical microscope image of amphotericin B-loaded hollow microneedle. The microneedles were purposely broken to show the hollow part of the needles. (C) blue-colored substrate for enhanced contrast of the amphotericin B-loaded microneedles. (D) colorless substrate of the amphotericin B-loaded microneedles.


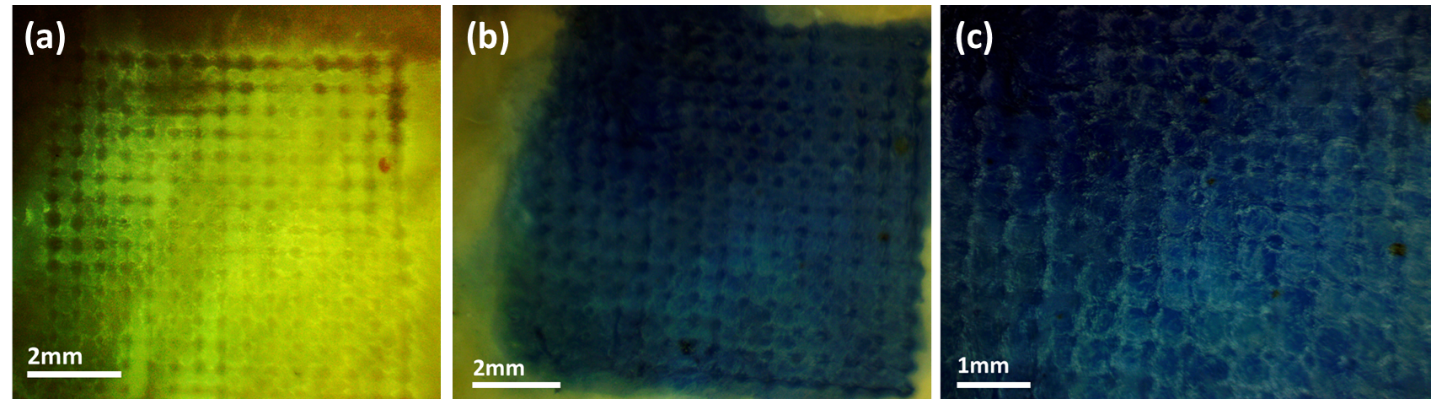


Fig. S3. Illustration of *ex vivo* skin penetration test. Optical images of hollow microneedle array penetrated porcine skin (A) before and ((B) and (C)) after treatment with Trypan Blue solution.
